# Flavonoids promote *Rhizophagus irregularis* spore germination and tomato root colonization: A target for sustainable agriculture

**DOI:** 10.3389/fpls.2022.1094194

**Published:** 2023-01-05

**Authors:** Javier Lidoy, Estefanía Berrio, Marta García, Luis España-Luque, Maria J. Pozo, Juan Antonio López-Ráez

**Affiliations:** Dept. of Soil Microbiology and Symbiotic Systems, Estación Experimental del Zaidín, Consejo Superior de Investigaciones Científicas (CSIC), Granada, Spain

**Keywords:** bioinoculants, symbiosis, rhizosphere signaling, plant microbe communication, arbuscular mycorrhiza (AM), flavonoids

## Abstract

The use of arbuscular mycorrhizal (AM) fungi has great potential, being used as biostimulants, biofertilizers and bioprotection agents in agricultural and natural ecosystems. However, the application of AM fungal inoculants is still challenging due to the variability of results when applied in production systems. This variability is partly due to differences in symbiosis establishment. Reducing such variability and promoting symbiosis establishment is essential to improve the efficiency of the inoculants. In addition to strigolactones, flavonoids have been proposed to participate in the pre-symbiotic plant-AM fungus communication in the rhizosphere, although their role is still unclear. Here, we studied the specific function of flavonoids as signaling molecules in AM symbiosis. For that, both *in vitro* and *in planta* approaches were used to test the stimulatory effect of an array of different subclasses of flavonoids on *Rhizophagus irregularis* spore germination and symbiosis establishment, using physiological doses of the compounds. We show that the flavone chrysin and the flavonols quercetin and rutin were able to promote spore germination and root colonization at low doses, confirming their role as pre-symbiotic signaling molecules in AM symbiosis. The results pave the way to use these flavonoids in the formulation of AM fungal-based products to promote the symbiosis. This can improve the efficiency of commercial inoculants, and therefore, help to implement their use in sustainable agriculture.

## 1 Introduction

The growing human population requires a considerable increase in food production, leading to overexploitation of natural resources (Godfray et al., 2010). Crop varieties with higher yields and greater resistance to environmental stresses and diseases are currently being developed. However, massive use of chemical fertilizers and pesticides is still required to provide essential nutrients and reduce disease damage in agricultural production systems. The use and abuse of these chemical products in agriculture have a huge environmental impact, polluting soils and aquifers and contributing to climate change, negatively affecting human health, ecosystems and species worldwide (Tilman et al., 2002; Evans et al., 2019; Lynch et al., 2021). Therefore, there is an urgent need to find more sustainable and environmentally friendly alternatives to reduce the use of these harmful agrochemicals (Geiger et al., 2010).

One strategy that is gaining momentum is the use of beneficial microorganisms with biostimulant properties. These microorganisms can establish symbiotic associations with plants improving agroecosystems and crop production (Tkacz and Poole, 2015). Among these beneficial microorganisms stand out arbuscular mycorrhizal (AM) fungi. These soil fungi belong to the phylum *Glomeromycota* and establish mutualistic associations with plant roots known as AM symbiosis (Smith and Read, 2008). AM symbiosis is about 450 million years old, and it is established with more than 70% of land plants, including most species of agronomic and industrial interest (cereals, vegetables, fruit trees, cotton, etc.), as well as ornamental and forest species (Barea et al., 2005; Brundrett and Tedersoo, 2018). It is characterized for the formation of specific structures within the roots of the host plant known as arbuscules (Parniske, 2008). In the arbuscules takes place the nutrient exchange between the fungus and the host plant (Bonfante and Genre, 2010). In addition to the arbuscules, the AM fungus develops a large network of hyphae, known as extraradical mycelium, which serves to explore larger areas of soil and constitutes the assimilative structure for mineral nutrients and water, functioning as pseudo roots (Parniske, 2008). The benefits of AM symbiosis in plant nutrition and health are well known (Barea et al., 2005; Wipf et al., 2019). However, in addition to a better nutrition, AM symbioses offer other benefits to the host plant including improved defense responses to pathogens and increased resilience to environmental stresses, such as drought and salinity (Pozo et al., 2015).

Despite the potential benefits of AM fungi, their application as biostimulants in agricultural settings is still challenging due to the variability of the results in production systems, which hinders their commercialization and implementation (Tkacz and Poole, 2015). This variability resides mainly in three factors: a) the quality and effectiveness of the inoculants, b) the environmental conditions and c) the management techniques, especially chemical fertilization. AM fungi are obligate biotrophs, so they depend on a host plant to develop and complete their life cycle (Parniske, 2008). This makes it difficult to implement the production of stable, axenic and homogeneous inoculants based on AM fungi. Spore-based inocula are available on the market, and they are easy to quantify and store, with higher homogeneity and lower risk of contamination than soil based inocula. However, spore production *in vitro* is costly (Siddiqui and Kataoka, 2011).

The establishment and functioning of AM symbiosis requires a high degree of coordination between the AM fungus and the host plant, based on precise molecular communication (Pozo et al., 2015; López-Ráez et al., 2017). The molecular dialogue is initiated early during the pre-symbiotic phase with the production and exudation into the rhizosphere of signaling molecules by the plant, primarily strigolactones (SLs) (López-Ráez et al., 2017). SLs are specifically recognized by the AM fungus present in the vicinity of the roots, stimulating spore germination, hyphal branching and exudation of fungal Myc-factors, thus facilitating the contact between the two partners and the establishment of the symbiosis (Akiyama et al., 2005; Besserer et al., 2006; Bonfante and Genre, 2010). SLs are derived from carotenoids and, according to their signaling role, they are produced at very low amounts by the plant (on the order of pico- and nanomolar), according to the plant’s nutritional status (López-Ráez et al., 2008; Yoneyama et al., 2012; Marro et al., 2022). In addition to signaling compounds in the rhizosphere, SLs are plant hormones regulating plant responses to nutritional stresses, especially phosphate (Pi) deficiency (Gomez-Roldan et al., 2008; Umehara et al., 2008; Marro et al., 2022).

**Figure 1:**
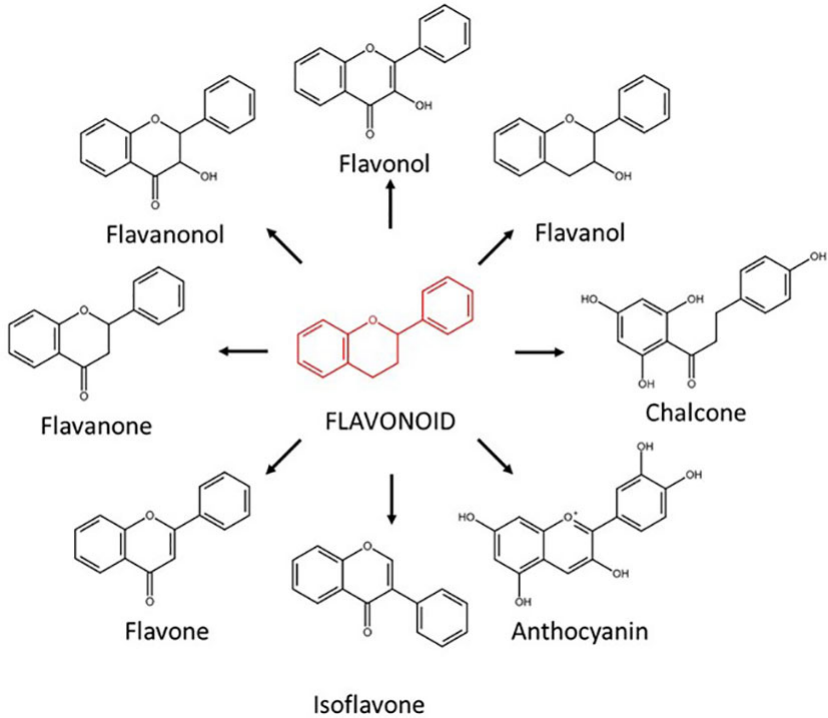
Schematic overview of the different groups of flavonoids according to their chemical structure.

In addition to SLs, other plant-derived compounds such as flavonoids have been proposed to participate in the pre-symbiotic molecular dialogue in AM symbiosis (reviewed in Hassan & Mathesius (2012)). However, the flavonoids specific role and functioning is not clear. Flavonoids comprise a large and diverse family of ubiquitous secondary metabolites belonging to the phenylpropanoids. They play a diverse array of biological functions in plants, acting as antioxidants, pigments in flowers, fruits and vegetables, regulators of auxin transport, fertility, defense barriers against herbivores and microbial pathogens (phytoalexins), regulating root architecture and as signaling compounds in beneficial plant-microbe symbioses in the rhizosphere (Hassan and Mathesius, 2012). So far, more than 10,000 different flavonoids have been characterized. According to their chemical structure, they are subcategorized into different major groups, including flavonols, anthocyanin, flavones, isoflavonoids, flavanonols, flavanones, flavanols, and chalcones (**Figure 1**) (Panche et al., 2016). Regarding their role as signaling molecules in the rhizosphere, the best-known function is associated to the *Rhizobium*-legume symbiosis (Singla and Garg, 2017). This beneficial symbiosis is established between legumes and certain rhizobacteria, leading to the fixation of atmospheric nitrogen and providing nitrogen to the host plant under nitrogen deficiency (Masson-Boivin and Sachs, 2018). The pre-symbiotic and symbiotic stages in the *Rhizobium*-legume symbiosis and AM symbioses are similar, and they share some of the required signaling components forming the so-called SYM pathway (Mukherjee and Ané, 2011; de Bruijn, 2020). In the Rhizobium-legume symbiosis, the molecular dialogue during the pre-symbiotic phase is initiated with the production and exudation into the rhizosphere of certain flavonoids (isoflavonoids) by the host plant (**Figure 1**). These isoflavonoids are involved in the recruitment of compatible rhizobia by inducing or inhibiting bacterial Nod factors (Shaw et al., 2006; Mandal et al., 2010).

The role of flavonoids in AM symbiosis is ambiguous and unclear. Initially, they were considered not important for AM establishment (Becard et al., 1995). Few years later, it was shown that certain flavonoids presented activity either stimulating spore germination or root colonization (Akiyama et al., 2002; Scervino et al., 2007; Steinkellner et al., 2007). However, the role of flavonoids in AM symbiosis is still controversial as positive, negative or neutral results have been described (Vierheilig et al., 1998; Singla and Garg, 2017). This controversy may be related to the very different experimental conditions used, as they study different flavonoids, different concentrations and different fungal genotypes (Vierheilig et al., 1998; Singla and Garg, 2017). Thus, the specific involvement and functioning of flavonoids in AM symbiosis remains unclear. We hypothesize that the exogenous application of flavonoids may enhance the effectiveness of AM inoculants by acting as signaling molecules during the pre-symbiotic phase of the AM symbiosis. Different flavonoids belonging to different subcategories and at different concentrations were tested, both *in vitro* and *in planta*, for their capacity to induce spore germination and stimulate root colonization by the AM fungus *Rhizophagus irregularis* (formerly *Glomus intraradices*), the most widely used AM fungus in commercial products in the market. The results confirm the bioactivity of these compounds in the symbiosis and reveal that there is class specificity and their activity depends on the dose used.

## 2 Material and methods

### 2.1 *In vitro* germination of spores of the AM fungus *R. irregularis*

The *in vitro* assays were carried out in 90 mm diameter Petri dishes with 35 ml of agar medium (2%) in deionized water under sterile conditions. The flavonoids used were the flavonols quercetin and rutin (Sigma-Aldrich, Germany), the flavone chrysin (Sigma-Aldrich, Germany), the isoflavone genistein (Sigma-Aldrich, Germany) and the pterocarpene medicarpin (kindly provided by Dr. Francisco A. Macías, University of Cádiz, Spain). As positive control, the active enantiomer of the synthetic SL analogue 2’-*epi*-GR24 (GR24^4DO^, StrigoLab, Italy) (Scaffidi et al., 2014) was used. For the preparation of the different treatments, stock solutions (1 mM) were prepared by dissolving the different compounds in 100% acetone. Serial dilutions in deionized water were prepared for each compound. Prior the addition to the Petri dishes, the solutions were sterilized using 0.22 µm filters. All treatments, including the controls, had a final concentration of acetone in the plate of 1‰. In a laminar flow hood, 50 µl of the corresponding dilution were added per plate and spread homogeneously over the entire agar surface using a seeding loop. The plates were kept open for 30 min to allow absorption of the added compounds and for acetone evaporation. Subsequently, a solution with 15 axenic spores of *R. irregularis* [MUCL 57021; kindly supplied by Koppert Biological Systems (The Netherlands)] were added per plate. Plates were sealed and incubated upside down at darkness at 25°C. Spore germination was evaluated daily. Due to the presence of multiple hyphae from the starter inoculum, germination was quantified by assessing the growth of new hyphae through the culture medium. Two independent experiments were performed with different concentrations of flavonoids, always within a physiological concentration range. For the experiment 1, 5 independent replicates per treatment were used [5 plates with 15 spores per plate; therefore (75 spores per treatment)]. For the experiment 2, 7 replicates per treatment [7 plates with 15 spores per plate (105 spores per treatment)] were used.

### 2.2 AM colonization *in planta*

Tomato (*Solanum lycopersicum* L.) seeds of the genotypes Red Cherry (LA0337), kindly provided by Dr. Gregg Howe (Michigan State University, USA) and Kardia (Syngenta, Spain) were surface sterilized with 50% commercial bleach for 10 min and after washed thoroughly with tap water. The seeds were then sown in sterilized vermiculite and incubated at 25–27°C, 16h/8h (day/night) and 65-70% relative humidity in a climatic chamber. Ten-day-old seedlings were transplanted individually into 100 ml growing cells with sterile sand:vermiculite (1:1). Plants were inoculated with spores of *R. irregularis* (MUCL 57021; Ri plants) supplied by Koppert Biological Systems (The Netherlands). 700 and 300 spores were used for the assay with the cultivar Red Cherry and Kardia, respectively. As mycorrhizal control, a set of non-inoculated plants was included (Nm plants). Ri plants were treated with quercetin, rutin, chrysin or genistein, at two different concentrations 0.01 and 0.1 µM. As a positive control, a treatment with the synthetic SL analogue GR24^4DO^ was included. Negative controls were also included with non-treated plants. For the application of the different compounds (flavonoids and GR24^4DO^), serial dilutions in Hewitt nutrient solution were prepared for each of the 1mM stock solutions prepared. Prior to their addition, the corresponding serial dilutions of the different compounds were prepared in Hewitt’s nutrient solution (Hewitt, 1953), at a final acetone concentration of 1‰. To favor mycorrhizal symbiosis establishment, modified Hewitt’s solution was used containing 25% of the standard phosphate levels (0.33 mM). Plants were treated twice a week with 10 ml of the different compound dilutions. The control (untreated) treatments were also irrigated twice a week with 10 ml of Hewitt solution containing 1‰ acetone. Ten independent replicates per treatment were used. Mycorrhizal levels were assessed 6 weeks after transplanting.

### 2.3 Quantification of mycorrhizal colonization

Quantification of mycorrhizal colonization was performed by histochemical staining as described in García et al. (2020). Briefly, roots were cleared and digested in a solution of 10% KOH (w/v) for 2 days at room temperature. The alkaline solution was washed thoroughly with tap water and acidified with a 2% (v/v) acetic acid solution. The fungal root structures were stained with a 5% (v/v) black ink (Lamy, Germany) and 2% acetic acid solution incubated at room temperature (Vierheilig et al., 2005). After 24h the ink was washed with water and colonization was determined by the gridline intersection method (Giovannetti and Mosse, 1980) using a Nikon SMZ1000 stereomicroscope.

### 2.4 Statistics

To identify significant differences between the means, statistical analyses were performed with unpaired t-test analysis using Statgraphics Plus 3.1. Since the percentage of germination and mycorrhizal colonization did not have a normal distribution, the Bliss transformation was applied to the data before the analysis.

## 3 Results

To deepen in the role of flavonoids as pre-symbiotic signals in AM symbiosis, the capacity of a series of flavonoids belonging to different subcategories of stimulating the germination of spores of the AM fungus *R. irregularis* was assessed *in vitro* (**Figure 1**). Different concentrations, within physiological levels, were used. Spores of *R. irregularis* were used in the experiments since most AM fungal commercial products are based on this fungus as biostimulant. Two independent experiments were assessed:

### 3.1 Stimulatory effect of flavonoids of AM symbiosis *in vitro*

**Figure 2:**
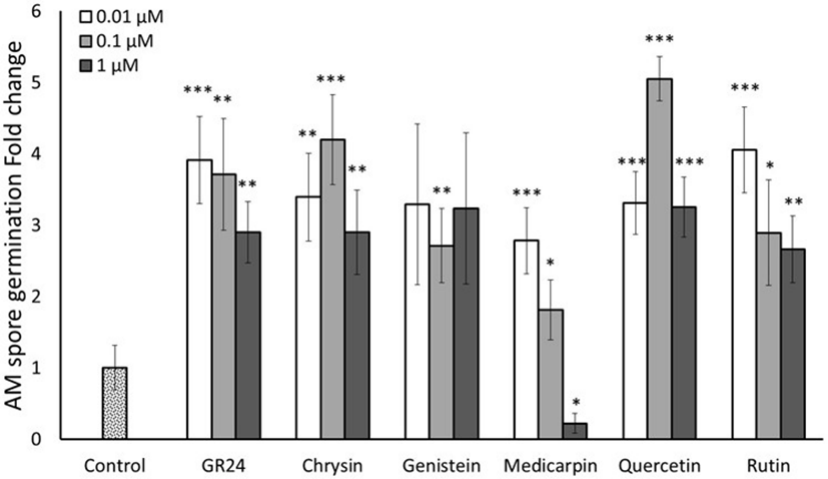
Effect of flavonoid treatments on *in vitro R. irregularis* spore germination. Relative percentage of germination in spores incubated for 10 days in Petri dishes with 2% agar medium with three different concentrations (0.01, 0.1 and 1 µM) of the flavonoids chrysin, genistein, medicarpin, quercetin and rutin. The synthetic strigolactone analogue GR24^4DO^ (GR24) was used as a positive control. The bars correspond to the mean of 5 independent replicates (15 spores per replicate) ± S.E. T-test analysis between each treatment compared with the control. *p<0.05, **p<0.01, ***p<0.001.

In a first assay, three different concentrations (0.01, 0.1 and 1 µM) of the different flavonoids were tested. SLs are well-known pre-symbiotic signals in AM symbiosis, having the ability to stimulate spore germination and hyphal branching of AM fungi (Akiyama et al., 2005; Besserer et al., 2006). Therefore, a treatment with an active enantiomer of the synthetic SL analogue 2’-*epi*-GR24 (GR24^4DO^) (Scaffidi et al., 2014) was included as a positive control. Spore germination was checked daily from the third day. Germination levels were quantified 10 days upon application. GR24^4DO^ induced spore germination at all three concentrations used, showing a slight decrease at the highest concentration (1 µM), Validating the bioassay and confirming the viability of the spores (**Figure 2**). The five flavonoids tested (genistein, medicarpin, chrysin, quercetin and rutin) also stimulated spore germination of *R. irregularis* compared to the control. Genistein induced about 2.5 times germination at all the concentrations tested (**Figure 2**). Medicarpin application stimulated spore germination 2.8- and 1.8-fold at the lower concentrations, 0.01 and 0.1 µM, respectively. Conversely, a significant inhibitory effect on spore germination was observed at the highest concentration (1 µM). For the flavone chrysin, the highest stimulation of germination was observed after application of 0.1 µM, with a 4.2-fold increase respect to the control. The flavonol quercetin stimulated spore germination at the three tested concentrations. The highest induction in germination was observed at 0.1 µM, with a 5-fold increase. Upon application of 0.01 and 1 µM, about 3 times induction was observed (**Figure 2**). Rutin also induced germination at the three concentrations tested, being the highest stimulation observed at the lowest concentration (0.01 µM), with about 4-fold increase respect to the control. At higher concentrations (0.1 and 1 µM), germination was stimulated 2.9 and 2.7 times, respectively (**Figure 2**).

**Figure 3:**
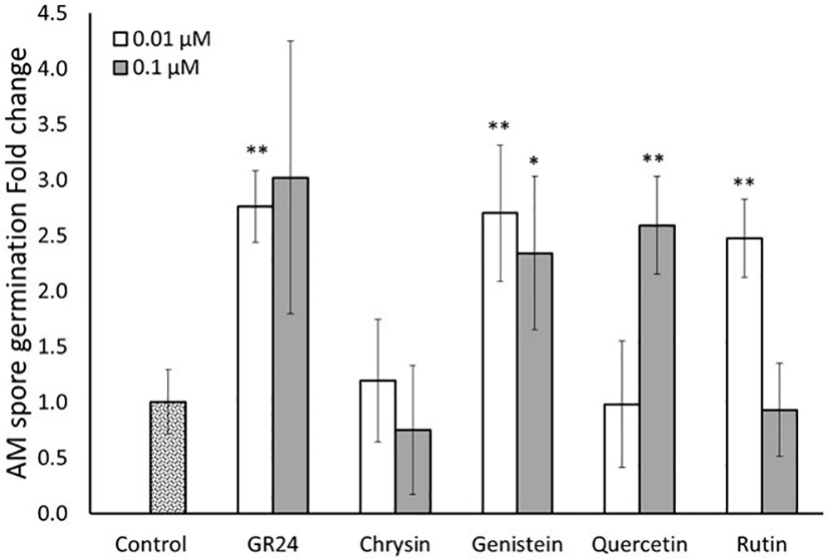
Effect of flavonoid treatments on *in vitro R. irregularis* spore germination. Relative percentage of germination in spores incubated for 5 days in Petri dishes with 2% agar medium with two different concentrations (0.01 and 0.1 µM) of the flavonoids chrysin, genistein, quercetin and rutin. The application of the synthetic strigolactone analogue GR24^4DO^ (GR24) was used as a positive control. The bars correspond to the mean of 7 independent replicates (15 spores per replicate) ± S.E. T-test analysis between each treatment compared with the control. *p<0.05, **p<0.01.

To confirm the results observed, a second spore germination assay *in vitro* was carried out. According to the previous results, only the lower concentrations (0.01 and 0.1 µM) were used for the different compounds in this second assay. Here, spore germination was faster than in the previous experiment and germination levels were quantified 5 days after application of the different compounds. As before, GR24^4DO^ induced spore germination at both concentrations used, again validating the bioassay and spore’s viability (**Figure 3**). In this experiment, only the four flavonoids that showed the higher effect on germination in the previous assay (chrysin, genistein, quercetin and rutin) were tested. No effect of the flavone chrysin was detected at any of the concentration used (**Figure 3**). In the case of the isoflavone genistein, both concentrations stimulated germination of the spores of *R. irregularis*. The application of 0.01 and 0.1 µM induced germination 2.7 and 2.3-fold, respectively, compared to the control (**Figure 3**). These inductions were similar to that observed for the positive control GR24^4DO^ (**Figure 3**). The flavonol quercetin promoted spore germination about 2.5 times compared to the control at 0.1 µM, while no stimulatory effect was observed at the lower concentration (0.01 µM) (**Figure 3**). In the case of rutin, a 2.5-fold promotion was observed at the lower concentration (0.01 µM), showing similar stimulation levels to those observed for GR24^4DO^ (**Figure 3**). No significant effect was detected at 0.1 µM. The results showed that certain flavonoids belonging to different subcategories, have the capacity of stimulate the germination of the spores of the AM fungus *R. irregularis in vitro* at low concentrations. Remarkably, the results also indicate that the effect is dose dependent.

### 3.2 Stimulatory effect of flavonoids on AM symbiosis establishment *in planta*

**Figure 4:**
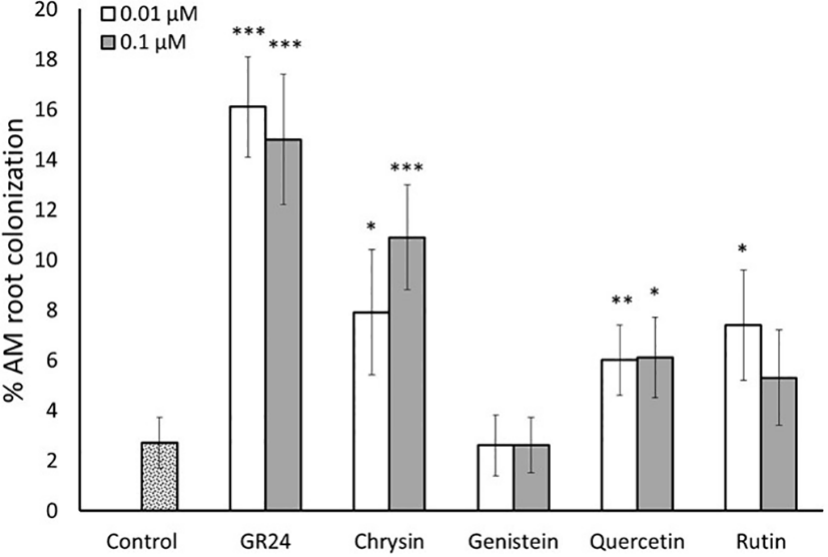
Root colonization of tomato plants by the mycorrhizal fungus *R. irregularis*. Plants were inoculated with *R. irregularis* spores and treated twice a week with two different concentrations (0.01 and 0.1 µM) of the flavonoids chrysin, genistein, quercetin and rutin. The synthetic strigolactone analogue GR24^4DO^ (GR24) was used as a positive control. The bars correspond to the mean of 10 independent replicates ± S.E. T-test analysis between each treatment compared with the control. *p<0.05, **p<0.01, ***p<0.001.

Based on the results obtained *in vitro*, we next carried out an *in planta* experiment to determine whether the increased spore germination rate induced by flavonoids resulted in higher mycorrhizal root colonization. Tomato (cv. Red Cherry) as a host plant and spores of the same *R. irregularis* strain (MUCL 57021) used in the *in vitro* assays were used. As expected, the application of GR24^4DO^ highly (about 6 times) enhanced mycorrhizal colonization levels of *R. irregularis* at 0.01 and 0.1 µM compared to control plants (**Figure 4**). Regarding the flavonoid treatments, no significant effect in mycorrhization was observed upon application of the isoflavone genistein at any of the two concentrations applied. Conversely, a stimulatory effect was observed for the other three compounds tested. The flavone chrysin induced mycorrhizal colonization levels about 3 and 4 times at 0.01 and 0.1 µM, respectively, compared to the control (**Figure 4**). The flavonol quercetin promoted mycorrhizal colonization more than 2 times after application of both 0.01 and 0.1 µM (**Figure 4**). The other flavonol, rutin, increased mycorrhization about 3-fold upon application of 0.01 µM and about 2-fold at 0.1 µM, although this increase was not statistically significant (**Figure 4**). The results show that the flavonoids chrysin, quercetin and rutin function as signaling molecules in the rhizosphere stimulating the establishment of AM symbiosis.

### 3.3 Quercetin promotes AM symbiosis in commercial tomato rootstocks

**Figure 5:**
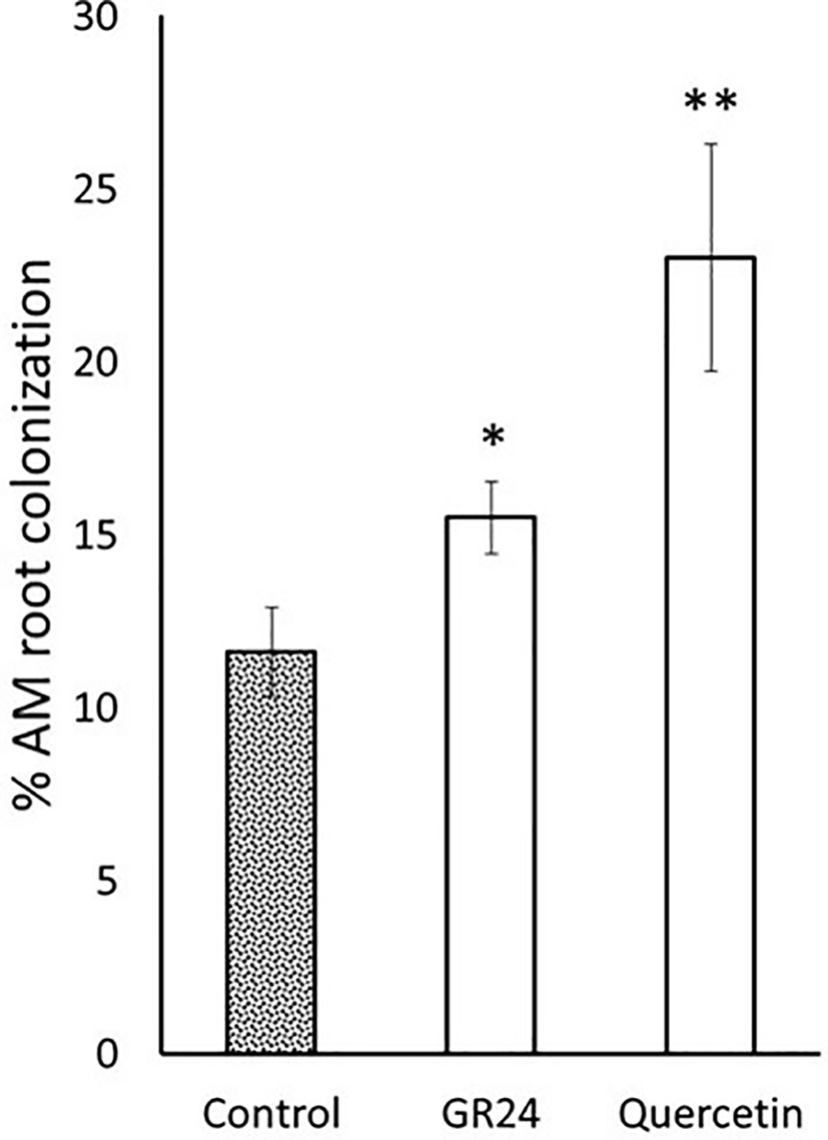
Mycorrhizal root colonization of rootstock tomato plants by the AM fungus *R. irregularis*. Plants were inoculated with *R. irregularis* spores and treated with quercetin (1 µM). The application of the synthetic strigolactone analogue GR24^4DO^ (GR24) was used as a positive control (0.1 µM). The bars correspond to the mean of 10 independent replicates ± S.E. T-test analysis between each treatment compared with the control. *p<0.05, **p<0.01.

Currently, the vast majority of tomato production is carried out using grafted plants (Raymond, 2013). Grafting is a horticulture technique that combine and use beneficial traits of both the rootstock and the scion plants. Hereto, a rootstock is selected for its resistance to soilborne pathogens and/or its ability to increase vigor and fruit yield. Then, the rootstock can be combined with different scions selected for their fruit quality characteristics. To further study the potential use of flavonoids in agriculture to improve AM fungal-based commercial products, a mycorrhizal experiment was carried out using the commercial tomato rootstock Kardia (Syngenta). The flavonol quercetin was selected because of the previous results and its reduced cost compared to the other flavonoids tested, which makes it more interesting from a commercial point of view. A slight increase of 1.3 times in mycorrhizal colonization was observed upon application of 0.1 μM GR24^4DO^ (**Figure 5**). Application of 1 µM quercetin promoted root colonisation by 2-fold compared to the untreated control plants (**Figure 5**), confirming the ability to stimulate AM symbiosis in different genotypes, including hybrid lines of great agronomic interest.

## 4 Discussion

In the present study, we carried out *in vitro* and *in planta* assays to confirm their involvement in this beneficial symbiosis with the aim of testing their potential use as additives to improve commercial AM fungal-based inoculants. The *in vitro* assays revealed that the flavonoids chrysin, genistein, medicarpin, quercetin and rutin, belonging to different subclasses, stimulated spore germination and hyphal growth of the AM fungus *R. irregularis* at different concentrations (**Figures 2**, **3**). They showed a stimulatory germination activity similar to that of the synthetic SL analogue GR24^4DO^, indicating their high and specific activity. A role for the flavone chrysin in AM fungal spore germination and hyphal development was previously described, although with contradictory results. First, an inhibitory effect on *Gi. margarita* was reported during the pre-symbiotic phase (Bécard et al., 1992; Chabot et al., 1992). Conversely, a stimulatory effect in the number of entry points and root colonization was later shown for *Gi. margarita*, *Funneliformis mosseae* and *R. irregularis* (Scervino et al., 2007). Therefore, the results seem to vary depending on the fungal genotypes, experimental conditions and, probably, the concentrations used, as this is crucial when using signaling compounds. Here, a stimulatory effect of chrysin was observed at low (‘physiological’, nanomolar range) doses, suggesting that this compound can act as a plant-derived signaling molecule during AM symbiosis establishment.

Our results are also consistent with the ability to stimulate spore germination and hyphal growth of the AM fungus *Gi. margarita in vitro* reported for certain flavonols, specially quercetin (Bécard et al., 1992; Chabot et al., 1992; Poulin et al., 1997; Scervino et al., 2005b). A role of quercetin in stimulating spore germination and hyphal growth has been reported also for other AM fungi, such as *Gi. rosea* (Scervino et al., 2005b) *and Gi. gigantea* (Baptista and Siqueira, 1997)*, F. mosseae* (Kape et al., 1993), *Claroideoglomus etunicatum* (Tsai and Phillips, 1991; Bécard et al., 1992)*, G. macrocarpum* (Tsai and Phillips, 1991) and *R. irregularis* (Bécard et al., 1992; Poulin et al., 1997). However, these effects were always observed at high concentrations (Vierheilig et al., 1998). Here, as for chrysin, we showed that quercetin is also able to stimulate fungal development at low concentrations (0.01 and 0.1 µM), supporting the role of flavonols a signaling molecules in AM symbiosis establishment. In agreement with this, a stimulatory effect in fungal development at low doses (0.01 µM) was also observed for rutin, a glycosylated derivative of quercetin. No effect in fungal development was previously described for rutin, although high concentrations of the compound were used in these experiments (Bécard et al., 1992; Chabot et al., 1992; Scervino et al., 2007). Once again, the different concentrations of the flavonoids tested could explain the divergences observed, since the dose is critical when working with signaling compounds.

Based on these and previous results, it is clear that certain flavonoids can stimulate AM fungal development during the pre-symbiotic phase of AM symbiosis *in vitro*. However, an effect *in vitro* does not necessarily correlate with an increased mycorrhizal colonization *in planta*. Remarkably, we show here the flavone chrysin, and the flavonols quercetin and rutin were also able to promote mycorrhizal colonization in tomato plants at low doses when applied in fertigation and using AM fungal spores as inoculum. This agrees with previous results in different plant species, including tomato. In tomato, the application of the flavones chrysin and luteolin, and the flavonol morin increased root colonization by different AM fungi, while other flavonols such as rutin, kaempferol and isorhamnetin showed no effect (Scervino et al., 2007). Quercetin was found to be present in mycorrhizal white clover (*Trifolium repens*) roots and shown to promote mycorrhizal colonization of *Gi. margarita* (Scervino et al., 2005a). Recently, quercetin has been related with the expansion of invasive plants (Pei et al., 2020; Tian et al., 2021; Borda et al., 2022). It was shown that these plants have increased levels of quercetin in their root exudates than native plants, which was associated to an enhanced mycorrhizal colonization and capacity of expansion. The authors also showed that the exogenous application of quercetin promoted AM fungal colonization of the target plants (Pei et al., 2020; Tian et al., 2021). The results suggest that the flavonol quercetin, and probably its derivatives such as rutin, act as signaling molecules in the rhizosphere promoting the establishment of AM symbiosis, as SLs do. Likely, both SLs and flavonols might act in tandem as ‘cry for help’ host signals to attract AM fungi and prepare the plant for colonization. In agreement with this idea, Maloney et al. (2014) proposed a role of flavonols, including quercetin, in the promotion of lateral root formation, which are the preferred place for the AM fungus to colonize the host plant. The results open up the possibility of using these compounds to improve the efficiency of commercial products based on AM fungal spores. Indeed, we show here that the addition of low doses of quercetin (at nanomolar levels) promote mycorrhizal colonization by *R. irregularis*, the most widely AM fungus used in commercial products. Remarkably, the effect seems to be not specific, as this assay was performed using two different tomato genotypes, including a tomato variety commonly used as rootstock. Most tomato farmers can benefit of this effect since currently the vast majority of tomato production is carried out using grafted plants (Raymond, 2013). Our findings support the use of this alternative strategy in tomato production, which could be extended to other crops produced in nursery conditions. However, further assays under field conditions should be performed before its implementation in production systems. Remarkably, most mycorrhizal plants, including crops with agronomic interest, produce these flavonoids, being probably sensitive to them. Therefore, this promoting effect of AM symbiosis could be extended to other crops.

Overall, we confirm here the role of flavonols in AM symbiosis and show their relevance as rhizosphere signaling molecules during the pre-symbiotic phase, promoting spore germination, hyphal development and symbiosis establishment. The increasing demand of AM fungal-based biostimulants in agriculture needs effective and efficient commercial inoculants, especially in seasonal crops. In this scenario, the addition of selected flavonoids -such as the flavone chrysin and the flavonol quercetin- at low doses has a great potential as accelerators of the pre-symbiotic phase, promoting symbiosis establishment and improving the efficiency of commercial products. The final goal of this research is the use these signaling compounds in agricultural production systems to implement the use of AMF as biostimulants, thus reducing the use of harmful agrochemicals. Remarkably, this management requires very reduced costs, which makes it achievable for most farmers. Therefore, this management has a great potential in sustainable agriculture. However, before its implementation we need first to confirm their effect in agricultural settings, as well as their effectiveness in different crops.

## Data availability statement

The raw data supporting the conclusions of this article will be made available by the authors, without undue reservation.

## Author contributions

JL and JAL-R contributed to the conception and design of the study. JL and MG performed the *in vitro* experiments. JL and EB performed the *in planta* bioassays. EB and LE-L quantified mycorrhizal colonization. JL performed the data analyses. JL and JAL-R drafted the manuscript and MJP revised it. All authors contributed to the article and approved the submitted version.

## References

[B1] Akiyama K. Matsuoka H. Hayashi H. (2002). Isolation and identification of a phosphate deficiency-induced c-glycosylflavonoid that stimulates arbuscular mycorrhiza formation in melon roots. Mol. Plant Microbe Interact. 15, 334–340. doi: 10.1094/MPMI.2002.15.4.334 12026171

[B2] Akiyama K. Matsuzaki K. Hayashi H. (2005). Plant sesquiterpenes induce hyphal branching in arbuscular mycorrhizal fungi. Nature 435, 824–827. doi: 10.1038/nature03608 15944706

[B3] Baptista M. J. Siqueira J. O. (1997). Efeito de flavonóides na germinacao e no crescimento assimbiótico de fungos micorrízicos vesículo-arbusculares. Braz. J. Plant Physiol. 6, 127–134.

[B4] Barea J. M. Pozo M. J. Azcón R. Azcón-Aguilar C. (2005). Microbial co-operation in the rhizosphere. J. Exp. Bot. 56, 1761–1778. doi: 10.1093/jxb/eri197 15911555

[B5] Bécard G. Douds D. D. Pfeffer P. E. (1992). Extensive *in vitro* hyphal growth of vesicular-arbuscular mycorrhizal fungi in the presence of CO(2) and flavonols. Appl. Environ. Microbiol. 58, 821–825. doi: 10.1128/aem.58.3.821-825.1992 16348673 PMC195340

[B6] Becard G. Taylor L. P. Douds D. D. Pfeffer P. E. Doner L. W. (1995). Flavonoids are not necessary plant signal compounds in arbuscular mycorrhizal symbioses. Mol. Plant-Microbe Interact. 8, 252–258. doi: 10.1094/MPMI-8-0252

[B7] Besserer A. Puech-Pagès V. Kiefer P. Gomez-Roldan V. Jauneau A. Roy S.. (2006). Strigolactones stimulate arbuscular mycorrhizal fungi by activating mitochondria. PloS Biol. 4, e226–e226. doi: 10.1371/journal.pbio.0040226 16787107 PMC1481526

[B8] Bonfante P. Genre A. (2010). Mechanisms underlying beneficial plant–fungus interactions in mycorrhizal symbiosis. Nat. Commun. 1, 48. doi: 10.1038/ncomms1046 20975705

[B9] Borda V. Reinhart K. O. Ortega M. G. Burni M. Urcelay C. (2022). Roots of invasive woody plants produce more diverse flavonoids than non-invasive taxa, a global analysis. Biol. Invasions 24, 2757–2768. doi: 10.1007/s10530-022-02812-8

[B10] Brundrett M. C. Tedersoo L. (2018). Evolutionary history of mycorrhizal symbioses and global host plant diversity. New Phytol. 220, 1108–1115. doi: 10.1111/nph.14976 29355963

[B11] Chabot S. Bel-Rhlid R. Chênevert R. Piché Y. (1992). Hyphal growth promotion *in vitro* of the VA mycorrhizal fungus, *Gigaspora margarita* becker & hall, by the activity of structurally specific flavonoid compounds under CO(2) -enriched conditions. New Phytol. 122, 461–467. doi: 10.1111/j.1469-8137.1992.tb00074.x 33874220

[B12] de Bruijn F. J. (2019). The common symbiotic signaling pathway (CSSP or SYM). Model. Legume Medicago truncatula 521–521. doi: 10.1002/9781119409144.part8

[B13] Evans A. E. Mateo-Sagasta J. Qadir M. Boelee E. Ippolito A. (2019). Agricultural water pollution: key knowledge gaps and research needs. Curr. Opin. Environ. Sustain 36, 20–27. doi: 10.1016/j.cosust.2018.10.003

[B14] García J. M. Pozo M. J. López-Ráez J. A. (2020). “Histochemical and molecular quantification of arbuscular mycorrhiza symbiosis,” in Plant and food carotenoids: Methods and protocols. Eds. Rodríguez-Concepción M. Welsch R. (New York, NY: Springer US), 293–299. doi: 10.1007/978-1-4939-9952-1_22 31745930

[B15] Geiger F. Bengtsson J. Berendse F. Weisser W. W. Emmerson M. Morales M. B.. (2010). Persistent negative effects of pesticides on biodiversity and biological control potential on European farmland. Basic Appl. Ecol. 11, 97–105. doi: 10.1016/j.baae.2009.12.001

[B16] Giovannetti M. Mosse B. (1980). An evaluation of techniques for measuring vesicular arbuscular mycorrhizal infection in roots. New Phytol. 84, 489–500. doi: 10.1111/j.1469-8137.1980.tb04556.x

[B17] Godfray H. C. J. Beddington J. R. Crute I. R. Haddad L. Lawrence D. Muir J. F.. (2010). Food security: the challenge of feeding 9 billion people. Science 327, 812–818. doi: 10.1126/science.1185383 20110467

[B18] Gomez-Roldan V. Fermas S. Brewer P. B. Puech-Pagès V. Dun E. A. Pillot J. P.. (2008). Strigolactone inhibition of shoot branching. Nature 455, 189–194. doi: 10.1038/nature07271 18690209

[B19] Hassan S. Mathesius U. (2012). The role of flavonoids in root–rhizosphere signalling: opportunities and challenges for improving plant–microbe interactions. J. Exp. Bot. 63, 3429–3444. doi: 10.1093/jxb/err430 22213816

[B20] Hewitt E. J. (1953). Sand and water culture methods used in the study of plant nutrition. Soil Sci. Soc. America J. 17, 301. doi: 10.2136/sssaj1953.03615995001700030033x

[B21] Kape R. Wex K. Parniske M. Görge E. Wetzel A. Werner D. (1993). Legume root metabolites and VA-mycorrhiza development. J. Plant Physiol. 141, 54–60. doi: 10.1016/S0176-1617(11)80851-5

[B22] López-Ráez J. A. Charnikhova T. Gómez-Roldán V. Matusova R. Kohlen W. de Vos R.. (2008). Tomato strigolactones are derived from carotenoids and their biosynthesis is promoted by phosphate starvation. New Phytol. 178, 863–874. doi: 10.1111/j.1469-8137.2008.02406.x 18346111

[B23] López-Ráez J. A. Shirasu K. Foo E. (2017). Strigolactones in plant interactions with beneficial and detrimental organisms: The yin and yang. Trends Plant Sci. 22, 527–537. doi: 10.1016/j.tplants.2017.03.011 28400173

[B24] Lynch J. Cain M. Frame D. Pierrehumbert R. (2021). Agriculture’s contribution to climate change and role in mitigation is distinct from predominantly fossil CO2-emitting sectors. Front. Sustain Food Syst. 4. doi: 10.3389/fsufs.2020.518039 PMC7116829 33644695

[B25] Maloney G. S. DiNapoli K. T. Muday G. K. (2014). The anthocyanin reduced tomato mutant demonstrates the role of flavonols in tomato lateral root and root hair development. Plant Physiol. 166, 614–631. doi: 10.1104/pp.114.240507 25006027 PMC4213093

[B26] Mandal S. M. Chakraborty D. Dey S. (2010). Phenolic acids act as signaling molecules in plant-microbe symbioses. Plant Signal Behav. 5, 359–368. doi: 10.4161/psb.5.4.10871 20400851 PMC2958585

[B27] Marro N. Lidoy J. Chico M.Á. Rial C. García J. Varela R. M.. (2022). Strigolactones: New players in the nitrogen–phosphorus signalling interplay. Plant Cell Environ. 45, 512–527. doi: 10.1111/pce.14212 34719040

[B28] Masson-Boivin C. Sachs J. L. (2018). Symbiotic nitrogen fixation by rhizobia–the roots of a success story. Curr. Opin. Plant Biol. 44, 7–15. doi: 10.1016/j.pbi.2017.12.001 29289792

[B29] Mukherjee A. Ané J. M. (2011). Germinating spore exudates from arbuscular mycorrhizal fungi: molecular and developmental responses in plants and their regulation by ethylene. Mol. Plant Microbe Interact. 24, 260–270. doi: 10.1094/MPMI-06-10-0146 21043574

[B30] Panche A. N. Diwan A. D. Chandra S. R. (2016). Flavonoids: an overview. J. Nutr. Sci. 5, e47–e47. doi: 10.1017/jns.2016.41 28620474 PMC5465813

[B31] Parniske M. (2008). Arbuscular mycorrhiza: the mother of plant root endosymbioses. Nat. Rev. Microbiol. 6, 763–775. doi: 10.1038/nrmicro1987 18794914

[B32] Pei Y. Siemann E. Tian B. Ding J. (2020). Root flavonoids are related to enhanced AMF colonization of an invasive tree. AoB Plants 12, plaa002. doi: 10.1093/aobpla/plaa002 32071712 PMC7015461

[B33] Poulin M. J. Simard J. Catford J. G. Librie F. Piché Y. (1997). Response of symbiotic endomycorrhizal fungi to estrogens and antiestrogens. Mol. Plant-Microbe Interact. 10, 481–487. doi: 10.1094/MPMI.1997.10.4.481

[B34] Pozo M. J. López-Ráez J. A. Azcón-Aguilar C. García-Garrido J. M. (2015). Phytohormones as integrators of environmental signals in the regulation of mycorrhizal symbiosesñ. New Phytol. 205, 1431–1436. doi: 10.1111/nph.13252 25580981

[B35] Raymond G. (2013). Grafting market developments. Rijk Zwaan USA, Salinas, CA, 26. Available at: http://www.vegetablegrafting.org/wp/wp-content/uploads/2013/11/session-4-raymond-scri-vege-grftg-symp-nov13.pdf.

[B36] Scaffidi A. Waters M. T. Sun Y. K. Skelton B. W. Dixon K. W. Ghisalberti E. L.. (2014). Strigolactone hormones and their stereoisomers signal through two related receptor proteins to induce different physiological responses in arabidopsis. Plant Physiol. 165, 1221–1232. doi: 10.1104/pp.114.240036 24808100 PMC4081333

[B37] Scervino J. M. Ponce M. A. Erra-Bassells R. Bompadre J. Vierheilig H. Ocampo J. A.. (2007). The effect of flavones and flavonols on colonization of tomato plants by arbuscular mycorrhizal fungi of the genera gigaspora and glomus. Can. J. Microbiol. 53, 702–709. doi: 10.1139/W07-036 17668030

[B38] Scervino J. M. Ponce M. A. Erra-Bassells R. Vierheilig H. Ocampo J. A. Godeas A. (2005a). Arbuscular mycorrhizal colonization of tomato by gigaspora and glomus species in the presence of root flavonoids. J. Plant Physiol. 162, 625–633. doi: 10.1016/j.jplph.2004.08.010 16008085

[B39] Scervino J. M. Ponce M. A. Erra-Bassells R. Vierheilig H. Ocampo J. A. Godeas A. (2005b). Flavonoids exclusively present in mycorrhizal roots of white clover exhibit a different effect on arbuscular mycorrhizal fungi than flavonoids exclusively present in non-mycorrhizal roots of white clover. J. Plant Interact. 1, 15–22. doi: 10.1080/17429140500192597

[B40] Shaw L. J. Morris P. Hooker J. E. (2006). Perception and modification of plant flavonoid signals by rhizosphere microorganisms. Environ. Microbiol. 8, 1867–1880. doi: 10.1111/j.1462-2920.2006.01141.x 17014487

[B41] Siddiqui Z. A. Kataoka R. (2011). “Mycorrhizal inoculants: Progress in inoculant production technology,” in Microbes and microbial technology: Agricultural and environmental applications. Eds. Ahmad I. Ahmad F. Pichtel J. (New York, NY: Springer New York), 489–506. doi: 10.1007/978-1-4419-7931-5_18

[B42] Singla P. Garg N. (2017). “Plant flavonoids: key players in signaling, establishment, and regulation of rhizobial and mycorrhizal endosymbioses,” in Mycorrhiza - function, diversity, state of the art. Eds. Varma A. Prasad R. Tuteja N. (Cham: Springer International Publishing), 133–176. doi: 10.1007/978-3-319-53064-2_8

[B43] Smith S. E. Read D. J. (2008). Mycorrhizal symbiosis (London: Academic Press). Available at: https://books.google.es/books?id=qLciOJaG0C4C.

[B44] Steinkellner S. Lendzemo V. Langer I. Schweiger P. Khaosaad T. Toussaint J. P.. (2007). Flavonoids and strigolactones in root exudates as signals in symbiotic and pathogenic plant-fungus interactions. Molecules 12, 1290–1306. doi: 10.3390/12071290 17909485 PMC6149470

[B45] Tian B. Pei Y. Huang W. Ding J. Siemann E. (2021). Increasing flavonoid concentrations in root exudates enhance associations between arbuscular mycorrhizal fungi and an invasive plant. ISME J. 15, 1919–1930. doi: 10.1038/s41396-021-00894-1 33568790 PMC8245413

[B46] Tilman D. Cassman K. G. Matson P. A. Naylor R. Polasky S. (2002). Agricultural sustainability and intensive production practices. Nature 418, 671–677. doi: 10.1038/nature01014 12167873

[B47] Tkacz A. Poole P. (2015). Role of root microbiota in plant productivity. J. Exp. Bot. 66, 2167–2175. doi: 10.1093/jxb/erv157 25908654 PMC4986727

[B48] Tsai S. M. Phillips D. A. (1991). Flavonoids released naturally from alfalfa promote development of symbiotic glomus spores *in vitro* . Appl. Environ. Microbiol. 57, 1485–1488. doi: 10.1128/aem.57.5.1485-1488.1991 16348488 PMC182973

[B49] Umehara M. Hanada A. Yoshida S. Akiyama K. Arite T. Takeda-Kamiya N.. (2008). Inhibition of shoot branching by new terpenoid plant hormones. Nature 455, 195–200. doi: 10.1038/nature07272 18690207

[B50] Vierheilig H. Bago B. Albrecht C. Poulin M. J. Piché Y. (1998). “Flavonoids and arbuscular-mycorrhizal fungi,” in Flavonoids in the living system. advances in experimental medicine and biology. Eds. Manthey J. A. Buslig B. S. (Boston, MA: Springer US), 9–33. doi: 10.1007/978-1-4615-5335-9_2 9781292

[B51] Vierheilig H. Schweiger P. Brundrett M. (2005). An overview of methods for the detection and observation of arbuscular mycorrhizal fungi in roots. Physiol. Plant 125, 393–404. doi: 10.1111/j.1399-3054.2005.00564.x

[B52] Wipf D. Krajinski F. van Tuinen D. Recorbet G. Courty P. E. (2019). Trading on the arbuscular mycorrhiza market: from arbuscules to common mycorrhizal networks. New Phytol. 223, 1127–1142. doi: 10.1111/nph.15775 30843207

[B53] Yoneyama K. Xie X. Kim H. Kisugi T. Nomura T. Sekimoto H.. (2012). How do nitrogen and phosphorus deficiencies affect strigolactone production and exudation? Planta 235, 1197–1207. doi: 10.1007/s00425-011-1568-8 22183123 PMC3362704

